# Fighting Noise Pollution: A Public Health Strategy

**DOI:** 10.1289/ehp.122-A58

**Published:** 2014-02-01

**Authors:** David C. Holzman

**Affiliations:** David C. Holzman writes on science, medicine, energy, economics, and cars from Lexington and Wellfleet, MA. His work has appeared in *Smithsonian*, *The Atlantic Monthly*, and the *Journal of the National Cancer Institute*.

In this issue of *EHP*, investigators from the University of Michigan at Ann Arbor describe the most serious health effects of noise and propose a blueprint for an effective U.S. public health response.^1^ The team, led by Richard L. Neitzel, an assistant professor of environmental health sciences, estimated that nearly one-third of Americans are exposed to noise levels deemed injurious to hearing by the U.S. Environmental Protection Agency (EPA)—a 24-hour average noise level exceeding 70 dBA.^1^ The authors based this figure on 1981 estimates from the EPA.^2^

But mounting evidence also connects noise exposures with cardiovascular disease, sleep disturbance, stress, general annoyance, impaired learning and concentration, and other health effects. The EPA’s limit for protecting against all health effects of noise is a 24-hour average 55 dBA, weighted with a penalty for nighttime exposures to account for the special impact of disrupted sleep.^2^ In 1981 the EPA estimated about 3% of the U.S. population could be exposed to noise levels high enough to increase the risk of hypertension.^2^

**Figure:**
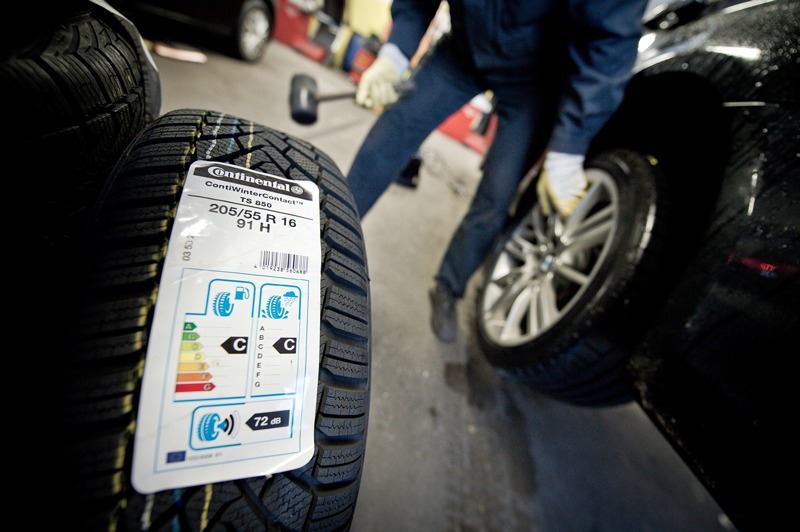
A recently implemented labeling requirement in Europe advises consumers about tires’ noise emissions as well as their energy economy and wet braking distance. © David Ebener/Corbis

Noise activates the body’s sympathetic nervous system, raising blood pressure and heart rate.^3^ Although inhabitants of noisy environments may be able to tune out noise, that habituation does not appear to extend to the cardiovascular system during nighttime exposures.^4^ Repeated arousals have been reported to prevent blood pressure from dropping during sleep the way it’s supposed to.^5^ Disrupted sleep is also associated with increased levels of lipids and the stress hormone cortisol, potentially increasing the risk of disorders such as depression^6^ and atherosclerosis.^7^

Two 2013 studies involving millions of British residents living airports reported an association between exposure to aircraft noise and increased hospital admissions for cardiovascular diseases.^8^^,^^9^ Annoyance can increase sympathetic tone—that is, put the sympathetic nervous system on high alert—especially in sensitive individuals.^10^ This increase in sympathetic tone has been postulated as a pathway by which individuals with high occupational noise exposures develop heart disease.”^11^

The major sources of chronic unwanted noise in the United States include road, rail, and air transportation,^12^ although adverse health effects are possible with enjoyable sources—loud sporting events, firearms, and music, for example.^1^ The authors advocate two approaches as the “least costly, most logistically feasible, and most effective” federal-level interventions: direct regulation of noise emissions, and improved public education. Between 1981 and 2000, regulation of aircraft noise under the Noise Control Act of 1972 drastically reduced the number of people exposed to excessive noise from neighboring airports, even though air travel increased sixfold in the same period.^13^ The EPA retains regulatory authority to reduce noise exposures from other sources, despite the 1981 defunding of its Office of Noise Abatement and Control. But the agency’s funding peaked in 1978, rendering action on noise control unlikely without congressional support, according to Neitzel and colleagues.^1^

Another strategy, labeling products with their noise emissions, is already mandatory for some products in the European Union, Argentina, Brazil, and China, and has been implemented in the United States for air compressors. The authors point out that labeling is only effective if consumers understand what they are reading; public education is critical.

The authors also recommend comprehensive noise mapping at the national level to identify hot spots for further study and remediation. The necessary data could be collected relatively inexpensively through crowdsourcing using people’s cell phones, Neitzel says: “We have a minicomputer in our pockets, every one with a microphone, and existing software platforms can compile noise data.” They also recommend sustainable building design and municipal procurement policies that consider noise levels when purchasing noisy items such as emergency vehicles, construction equipment, and buses.

Experts studying community noise highlight the need for controlling noise at the source. Arline Bronzaft, an environmental psychologist and professor emerita of City University of New York, cautions that communities that are quiet today “could be intruded upon in the near future.” It’s therefore important to focus on quieting communities overall, not just separating people from noise today, she says.

Others point out the need to consider noise as part of a holistic approach to public health. As one example, Hugh Davies, an associate professor in the School of Population and Public Health at the University of British Columbia, says efforts to mitigate traffic emissions must be achieved in ways that don’t increase noise levels. Distancing houses from vehicles reduces exposures to both noise and vehicular emissions, but reducing air pollution by reducing stop-and-go traffic may actually increase noise, because faster traffic is often noisier. Thus, Davies say, “we are replacing one health hazard with another.”
